# Colds

**Published:** 1903-04-11

**Authors:** 


### COLDS.

Dr. Walsh,[1] in dealing with this question, remarks that the word "cold," as applied to a complaint, is probably the most used and least understood in the practice of medicine. While it is supposed that low temperature has much to do with the process known as catching cold, most people are well aware that those who live in a dry, very cold climate are not so subject to colds as those who live in a moist, changeable one. It cannot be said either that changes of temperature are the important etiological element, for there are somewhat extensive daily variations of temperature in the arctic regions where colds are uncommon. The old term "rheum" was a better name for the affection, denoting as it does an excess of secretion. An ordinary cold represents a reaction of mucous membranes with a consequent increased production of mucus, with which is usually associated serum from the blood. In order for this excess of secretion to occur hyperæmia is necessary, and this is brought about either by the presence of some local irritant or by some disturbance of the circulation that causes passive congestion of the part. Over a century ago Erasmus Darwin showed that when portions of the surface of an animal's body are made cold and kept so for prolonged periods, there is a loss of tone in the blood-vessels of the part with consequent dilatation, and the circulation becomes very much slowed. Even after external warmth is restored to the parts a persistent paralytic dilatation of the blood vessels remains. In this way cold may have a share in setting up catarrh, but in the main microbes must be considered as the causation agents. The ordinary sequence may be this that some factor causes congestion of the nasal mucosa, which swells and so partially blocks up the nasal cavity, and the consequent failure of ventilation soon leads to the collection of secretion in which micro-organisms rapidly multiply. The tissues react to this microbic invasion, and there is a free flow of serum in which numbers of bacteria are carried off. It is evident, therefore, that at the commencement of a cold nothing should be done to prevent this flow of serum. The breathing in of microbe-laden air will assist in bringing about catarrh of the upper respiratory passages, but it is for affections of the mucous membrane below the larynx that the presence of bacteria in the breathed air is most important. It will be usually found that an attack of bronchitis or of pneumonia was preceded by a visit to some place in which there was a large crowd of people—a theatre, a church, a political meeting or some such assembly. Dr. Walsh calls attention in passing to the fact that theatres, churches and public halls are often kept badly dusted while they are not constructed so that their interiors may be properly exposed to the action of sunlight. A very striking indication that an ordinary cold is due to microbic action is to be found in the fact that the process is nearly always accompanied by fever. This is due to an absorption of bacillary toxins into the circulation resulting in a disturbance of the mechanism of heat regulation. The presence of fever is indicated by chilly feelings. Very often patients are sure that this is the beginning of a cold, so called, though in reality a little inquiry will usually elicit the fact that they have felt uncomfortable for some 48 hours previously. A distinct incubation period can be traced, and the efficient cause of the illness is commonly farther off than the patient imagines. The occurrence of fever and chilliness gives an indication for the first step in treatment. Since there are toxic materials in the blood, the eliminating organs of the body must be made active. Diaphoretics, diuretics, and purges are all of use. The old custom of giving calomel and a hot drink, especially hot cream of tartar lemonade, is almost sure to make the patient more comfortable. Great harm is undoubtedly done by the routine prescription of remedies containing opium.

[1] New York Medical News, March 14.

